# Alexander Carr

**Published:** 1905-03

**Authors:** 


### ALEXANDER CARR, M.R.C.S.

We regret to have to record the death, at the age of 70, of Mr. Alexander Carr, M.R.C.S., of Knowle, on January 23rd, 1905, one of our original members. Mr. Carr received his medical education at Charing Cross Hospital, where he was a favourite pupil of Charles Guthrie, then President of the Royal

College of Surgeons. He qualified as M.R.C.S. in 1854, and soon afterwards was appointed house-surgeon at the Westminster and afterwards at the Royal Westminster Ophthalmic Hospital, where he gave universal satisfaction. He fully intended to take up eyes as a speciality, and with that intention settled for a time in Park Lane and studied for his Fellowship. Financial losses, however, compelled him to go into general practice, and for the last forty years of his life he lived at Knowle.

He was a man universally respected, and he was associated with most of the social functions in the district. He was a staunch Conservative, and for a short time was one of the members of the old School Board. He was also a prominent High Churchman, and a regular attendant at Holy Nativity, Knowle, ever since it was built. Quite lately he had been elected vice-president of the Bristol Church Guild Union. Of a quiet, retiring disposition, he made no enemies, and his patients were essentially his friends. He was generous, and the poor of the district will sadly miss his kindness and gentleness.

He seldom spoke in public, but when he did so had a quaintness and dignity which always fixed the attention of his audience. Among other reminiscences, he used to relate that in pre-antiseptic days after amputating a leg in a colliery district he freely dusted the wound with pepper to keep off the vermin.

The funeral, at his own desire, was of the simplest character, and the church was crowded with acquaintances and friends.

Since Mr. Carr left London he gave up writing to periodicals, but previously he had written "Observations on the Pathology and Treatment of Mesenteric Disease," and had contributed to the *Lancet*, 1858, "On Application of Tincture of Iodine in Treatment of Trichiasis, Distichiasis, and Ptosis" and "Observations on the Treatment of Nebula."

